# Metagenomic and Metabolomic Analysis of Intestinal Excrement Differences Between Natural Hatching and Artificial Peeling out of the Shell in *Nipponia nippon*

**DOI:** 10.3390/ani16101472

**Published:** 2026-05-11

**Authors:** Guoqiang Qiu, Hongqing Bai, Jian Shi, Yu Xue, Tao Wang, Shidi Qin, Xiaolong Zhou, Ke He

**Affiliations:** 1 Deqing County Ecological Forestry Comprehensive Service Center, No. 101 Qianqiu East Street, Huzhou 313200, China; qiuguoqiang7777@163.com (G.Q.);; 2 Deqing County Bureau of Natural Resources and Planning, No. 101 Qianqiu East Street, Huzhou 313200, China; 3 Zhejiang Province Crested Ibis Rescue and Conservation Base, Xiazhu Lake National Wetland Park, Huzhou 313200, China; 4 Key Laboratory of Applied Technology on Green-Eco-Healthy Animal Husbandry of Zhejiang Province, College of Animal Science, Technology College of Veterinary Medicine, Zhejiang A&F University, 666 Wusu Road, Hangzhou 311300, China

**Keywords:** *Nipponia nippon*, metagenom, artificial shell peeling, metabolomics, intestinal health

## Abstract

The *Nipponia nippon* is a critically endangered species, and its breeding efforts are of vital importance for its conservation. Although artificial shell removal is sometimes employed in current breeding programs to increase survival rates, it may also have unknown impacts on chicks’ development. To investigate the influence of artificial shell removal on the gut microbiota composition in *Nipponia nippon*, metagenomic sequencing and untargeted LC-MS/MS analyses were performed. Samples from the early, mid, and late stages of natural hatching (ZE, ZM, ZL) and artificial shell removal (RE, RM, RL) were compared. Results indicated that the natural hatching groups formed a unique, highly diverse, and stable community by the late stage (ZL). Conversely, artificial peeling caused the microbial community succession to stagnate at an intermediate state. The RL group experienced a sharp decline in alpha diversity and a significant enrichment of opportunistic pathogens, such as *Edwardsiella*, *Clostridium*, and *Fusobacterium*. Functionally, the microbial community in the RL group remained in a stage of expanding basic functions rather than reaching an advanced equilibrium state. Metabolomic analysis confirmed this developmental arrest, revealing abnormal accumulations of organic acids, such as citric acid, and indole derivatives in the RL group. This indicates metabolic dysregulation, stress, and altered microbial–host chemical signaling. Furthermore, the significant biomarker *Edwardsiella* was strongly correlated with multiple differential metabolites in the RL group. Ultimately, these results indicate that artificial peeling intervention disrupts environmental adaptation and induces metabolic alterations in the intestinal development of the *Nipponia nippon* chicks.

## Simple Summary

This research investigated the effects of artificial eggshell removal during hatching on the gut health of *Nipponia nippon* chicks. Metagenomic and metabolomic sequencing were employed to compare naturally hatched chicks with those subjected to artificial shell removal across different developmental stages. The findings revealed that artificial shell removal inhibits the normal development of beneficial gut bacteria, leading to a loss of microbial diversity and a significant increase in pathogenic taxa. Furthermore, this intervention disrupts the chicks’ metabolic homeostasis, ultimately causing developmental delays and intestinal stress.

## 1. Introduction

The crested ibis (*Nipponia nippon*) is a flagship species for global avian conservation. Once widespread across East Asia, the species was considered extinct until the 1981 rediscovery of a remnant population of seven individuals in Shaanxi Province, China. While Shaanxi remains the primary stronghold, Zhejiang Province has become pivotal for the species’ range expansion. Central to this effort is Deqing County, where ten individuals (five breeding pairs) were introduced from Shaanxi in 2008 to re-establish the population in the lower Yangtze River region. The Xiazhu Lake Wetland, characterized by a mosaic of islands and abundant aquatic prey (loaches, eels, and frogs), provided an ideal habitat for acclimatization. This reintroduction program has achieved significant success; from the initial ten founding individuals, the population in Deqing has expanded significantly [1,2]. By late 2022, the local population exceeded 600 individuals, with nearly half reintegrated into the wild. Currently, Deqing represents the largest population in China and the largest in the eastern coastal region.

In the conservation of endangered species, maximizing the recruitment rate of every viable embryo is critical. In captive breeding programs, hatching failure—where fully developed embryos fail to externalize—remains a significant bottleneck. This mortality is often attributed to embryonic malpositioning, insufficient muscular vigor, or desiccation of the shell membranes due to humidity fluctuations. To mitigate these losses, conservationists employ Assisted Hatching (AH), a precision intervention involving the manual removal of the eggshell to facilitate emergence. In artificial incubation, “hatchability” often underperforms compared to natural incubation. Primary etiologies for hatching failure in crested ibises include the following: (1) malpositioning, where the embryo fails to align with the air cell, preventing the use of the egg tooth for pipping; (2) sticky chick syndrome, where hygroscopic imbalances cause the chorioallantoic membrane (CAM) to adhere to the down feathers; and (3) excessive shell thickness resulting from dietary supplementation in captive founders [3,4].

Assisted hatching is a multi-stage physiological intervention typically performed over 24–48 h. Stage 1 (Assessment): Embryos are monitored for “internal pipping” (vocalization or movement within the air cell). If external pipping does not occur within a 24 h window, intervention is indicated. Stage 2 (Ventilation): A small aperture is created at the air cell pole to alleviate hypoxia and stabilize oxygen saturation. Stage 3 (Vascular Assessment): This critical step involves inspecting the inner membrane. If the allantoic vasculature remains functional (active pulsation), the procedure is suspended to avoid hemorrhage and ensure proper yolk sac internalization. Membranes are maintained with sterile saline to prevent desiccation. Stage 4 (Facilitated Emergence): Once the vasculature has regressed, the shell is removed circumferentially. While the head is gently guided, the chick is encouraged to complete the exit through its own muscular effort to promote physiological tone [4,5,6].

Hatching failure represents a critical constraint on the population recovery of crested ibis [7]. Late-stage embryonic mortality—often precipitated by malpositioning, insufficient muscular tone, or adverse microclimatic conditions—necessitates the implementation of artificial shell-peeling technology (assisted hatching). This intervention serves as a requisite salvage strategy, mechanically facilitating emergence when natural pipping is obstructed. This project utilizes both naturally hatched and manually disassembled hatched crested ibis, and conducts metagenomic and metabolomic studies to investigate the effects of different hatching methods on the intestinal flora and digestive absorption functions of the cranes. Research into artificial shell-peeling technology (assisted hatching) for the crested ibis is significant because it directly addresses the “final mile” problem in captive breeding: the gap between successful fertilization and successful recruitment (survival).

## 2. Materials and Methods

### 2.1. Materials and Experimental Design

Fresh fecal samples were collected from 24 crested ibis chicks (*N* = 12 naturally hatched; *n* = 12 via assisted hatching) at the Xiazhu Lake Crested Ibis Breeding Center (Huzhou, China). Chicks aged 3 to 33 days were housed individually in 24 separate cages, each equipped with an independent feeding trough, allowing for unrestricted movement. All birds were provided with diets of identical composition throughout the study period. The samples were categorized into six groups based on developmental stage and hatching method: natural hatching groups: ZE (early-stage, 3–8 days), ZM (mid-stage, 13–18 days), and ZL (late-stage, 23–33 days); assisted hatching groups: RE (early-stage, 3–8 days), RM (mid-stage, 13–18 days), and RL (late-stage, 23–33 days). To ensure safety and sterility, the chicks were stabilized by experienced handlers while researchers collected swab samples using sterile equipment.

### 2.2. Microbial Diversity Analysis

Genomic DNA Library Construction and Sequencing. Genomic DNA (1 μg) was stochastically fragmented to an average size of 350 bp using a Covaris ultrasonic disruptor. Sequencing libraries were constructed following standard protocols, including end repair, A-tailing, adapter ligation, purification, and PCR amplification. Library integrity and insert size were verified via Automated Capillary Electrophoresis (AATI) analysis, and the effective concentration (>3 nM) was quantified using qPCR. Qualified libraries were pooled based on target data outputs and sequenced on an Illumina platform using a paired-end 150 bp (PE150) strategy. The raw sequencing reads have been deposited in the NCBI Sequence Read Archive (SRA) database (BioProject Accession Number: PRJNA1406017).

Gene Catalogue Construction and Taxonomic Annotation. The CD-HIT software (version 4.8.1, CRBS, San Diego, CA, USA) was utilized to eliminate redundancy from the ORF predictions, creating a non-redundant initial gene catalog. Clean reads from each sample were re-aligned to this catalogue using Bowtie2 v.2.5.5. Genes with a count of two or fewer reads were filtered out to finalize the unigene catalogue. Gene abundance was calculated based on aligned read counts and normalized by gene length. For taxonomic classification, unigenes were aligned against the Micro_NR database (comprising bacteria, fungi, archaea, and viruses) using DIAMOND v2.1.18. The lowest common ancestor (LCA) algorithm was applied to assign taxonomic annotations. Taxonomic abundance and diversity were subsequently analyzed using dimensionality reduction methods (PCA, PCoA, NMDS), community difference testing (ANOSIM), and biomarker discovery tools (including MetaGenomeSeq v.1.22.0, LEfSe v.1.1.2, and Random Forest models).

Functional profiling was conducted by aligning unigenes against multiple comprehensive databases—including KEGG, eggNOG, CAZy, VFDB, and PHI—using DIAMOND. The Best Hit (highest score) was selected to calculate relative functional abundances. Statistical analyses, such as PCA, NMDS, and LEfSe, were applied to assess functional divergence between groups. Finally, antimicrobial resistance was analyzed by aligning unigenes to the CARD database using the Resistance Gene Identifier (RGI). Mobile genetic elements (MGEs) were profiled by comparing sequences against the ISfinder, INTEGRALL, and plasmid databases to quantify MGE abundance for heatmap visualization. Functional and Resistance Gene Annotation. Functional profiling was conducted by aligning unigenes against multiple comprehensive databases—including KEGG, eggNOG, CAZy, VFDB, and PHI—using DIAMOND. The best BLAST hit (version 2.16.0, NCBI, Bethesda, MD, USA) was selected to calculate relative functional abundances. Statistical analyses, such as PCA, NMDS, and LEfSe, were applied to assess functional differences between groups.

### 2.3. Metabolome Profiling Analysis

Fecal samples of crested ibis (50 mg) were transferred to 2 mL centrifuge tubes equipped with 6 mm diameter grinding beads. Metabolite extraction was performed by adding 400 μL of an extraction solvent (methanol:water = 4:1, *v*/*v*) containing 0.02 mg/mL of L-2-chlorophenylalanine as an internal standard. Samples were homogenized using a Wonbio-96c frozen tissue grinder (Shanghai Wanbo Biotechnology Co., Ltd., Shanghai, China) for 6 min (at −10 °C, 50 Hz), followed by low-temperature ultrasonic extraction for 30 min (at 5 °C, 40 kHz). Subsequently, the samples were incubated at −20 °C for 30 min and centrifuged for 15 min (at 4 °C, 13,000× *g*). The resulting supernatant was transferred to autosampler vials for LC-MS/MS analysis. Raw data preprocessing was conducted using Progenesis QI software (V3.0, Waters Corporation, Milford, CT, USA). Metabolites were identified by querying the Human Metabolome Database (HMDB; http://www.hmdb.ca/, accessed on 12 December 2025), METLIN (https://metlin.scripps.edu/, accessed on 13 December 2025), and the Novogene Database. All raw data were deposited in the CNCB GSA database (accession number OMIX014467).

### 2.4. Statistical Analyses

Metagenomic Bioinformatics Analysis. Raw Illumina sequencing data were preprocessed using fastp to generate clean reads. Specifically, reads containing adapter contamination, excessive ambiguous nucleotides (>10%), or a high proportion of low-quality bases (>50%) were filtered out. To mitigate host DNA interference, clean reads were aligned to the *Nipponia nippon* reference genome using Bowtie2, and all host-derived sequences were discarded. The remaining high-quality reads were assembled into contigs using MEGAHIT v5. Open reading frames (ORFs) were predicted for sequences ≥ 500 bp using MetaGeneMark (version 3.38, Atlanta, GA, USA), and predicted ORFs shorter than 100 nt were excluded from subsequent analyses.

Metabolomic Analysis. The processed data matrix was uploaded to the Novogene Cloud Platform (https://magic-plus.novogene.com/#/tool/list, accessed on 18 December 2025) for comprehensive statistical analysis. Initial preprocessing involved retaining metabolic features detected in at least 80% of samples within any experimental group. For samples with metabolite levels below the lower limit of quantification, missing values were imputed using the minimum detected value. To account for variations in sample preparation and instrument instability, peak intensities were normalized using the sum normalization method. Furthermore, variables in Quality Control (QC) samples with a relative standard deviation (RSD) > 30% were excluded. The remaining variables were log10-transformed to stabilize variance for downstream analysis. Multivariate statistical analyses, including Principal Component Analysis (PCA) and Orthogonal Partial Least Squares Discriminant Analysis (OPLS-DA), were performed using the R package “ropls” (Version 1.6.2). Model stability was validated using 7-fold cross-validation. Significantly different metabolites (SDMs) were identified based on a Variable Importance in Projection (VIP) score > 1 from the OPLS-DA model and a *p*-value < 0.05 from Student’s *t*-test. These SDMs were mapped to biochemical pathways using the KEGG database (http://www.genome.jp/kegg/, accessed on 19 December 2025). Pathway enrichment analysis was conducted using the Python scipy.stats package (version 1.14.1, USA) to identify the biological pathways most relevant to the experimental treatments.

## 3. Results

### 3.1. Gut Microbial Composition and Diversity

**Figure 1:**
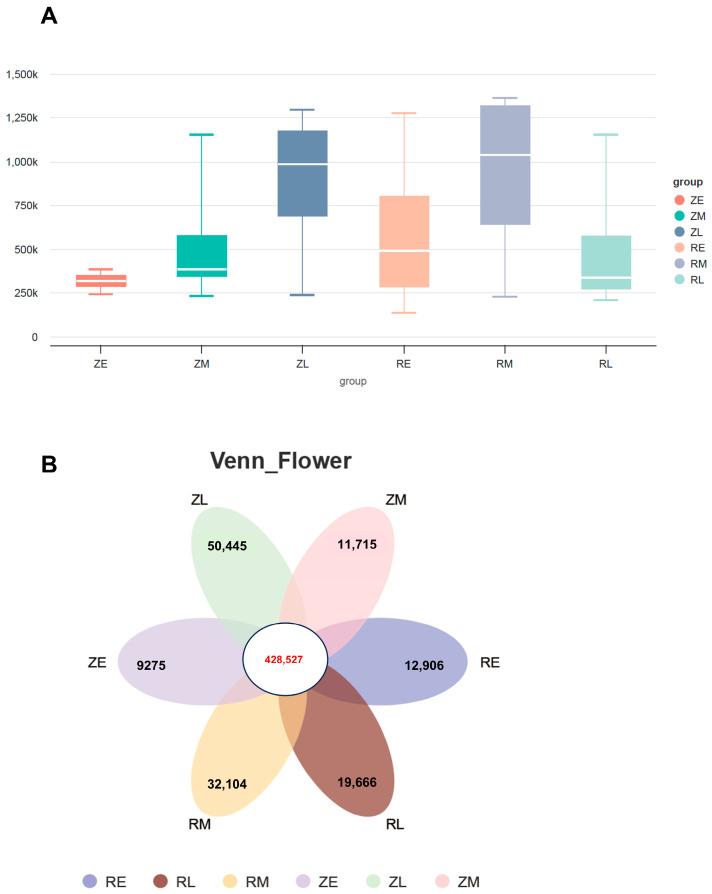
Analysis of gene amount differences in metagenomics: (**A**) The difference in the number of genes between the artificially shelled group and the naturally hatched group (*N* = 4). ZE (3–8 days after birth) = early stage of natural hatching; ZM (13–18 days after birth) = mid-stage of natural hatching; ZL (23–33 days after birth) = late-stage of natural hatching; RE (3–8 days after birth) = early-stage of artificial shell removal; RM (13–18 days after birth) = mid-stage of artificial shell removal, RL (23–33 days after birth) = late-stage of artificial shell removal); and (**B**) The common and unique genetic information among different groups was plotted using a Venn diagram or a petal diagram.

To investigate the influence of assisted hatching on the gut microbiota in crested ibis, metagenomic sequencing was performed to analyze the gene catalog size across groups. In the natural hatching cohort, the microbial community exhibited a progressive accumulation of genes from the ZE to ZL stages. Notably, a substantial expansion in gene numbers occurred between the ZM and ZL groups, with the median value increasing from approximately 400,000 to 1,000,000. This trend indicates that under natural conditions, the intestinal microbiota becomes increasingly complex over time, with succession reaching a highly diverse and stable climax community. In contrast, the assisted hatching groups (RE to RL) showed a non-linear trend in gene richness. Although gene numbers peaked in the RM group (median ~1,050,000), they declined sharply in the RL group, returning to a lower level. This reduction suggests that the RL group may have experienced intensified environmental selection pressure during the later developmental stage, leading to the extirpation of numerous species and a stochastic shift toward a less diverse community (Figure 1A). Consequently, gene richness was markedly higher in the ZL group than in the RL group; the former maintained high alpha diversity, whereas the latter exhibited a significant loss of microbial complexity. These results suggest that the ontogeny of intestinal flora diversity is severely disrupted by assisted hatching interventions. The Venn diagram illustrates the distribution of common and unique unigenes across the experimental groups. The number of unique genes in the ZE, ZM, ZL, RE, RM, and RL groups was 9275, 11,715, 50,445, 12,906, 32,104, and 19,666, respectively (Figure 1B).

**Figure 2:**
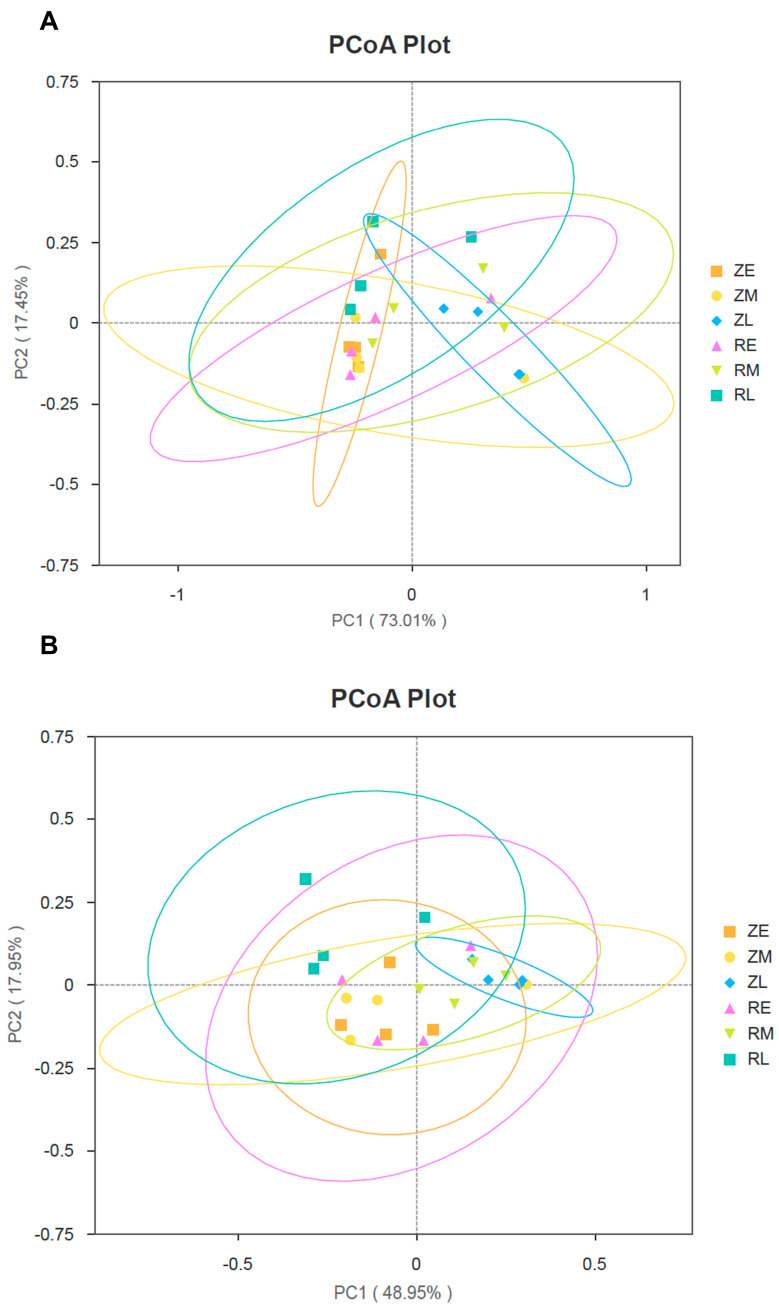
β-diversity of fecal microbiota in crested ibis: (**A**) PCoA analysis at the phylum level. ZE = Early-stage of natural hatching; ZM = Mid-stage of natural hatching; ZL = Late-stage of natural hatching; RE = Early-stage of artificial shell removal; RM = Mid-stage of artificial shell removal, RL = Late-stage of artificial shell removal); and (**B**) PCoA analysis at the genus level.

PCoA was performed at the phylum level to evaluate the dissimilarity in microbial community structure between groups. The first two principal components captured over 90% of the total variance, indicating that the ordination successfully represented the majority of the information within the dataset. In PCoA, the spatial proximity between points reflects the similarity in taxonomic composition (both richness and abundance), while increased distance indicates greater divergence. During the late developmental stage, the community structure of the natural hatching group (ZL) underwent a fundamental shift. Coupled with the previously observed peak in gene richness, these results indicate that the ZL group not only possessed the highest genetic diversity but also harbored a distinct taxonomic assembly compared to all other groups. This suggests the formation of a unique, highly diverse, and stable climax community under natural conditions. In contrast, the early and middle stages (ZE, ZM, RE, and RM) exhibited high similarity in phylum-level composition, suggesting a shared successional baseline regardless of the hatching method. However, the RL group showed extreme inter-individual variation and a lack of community clustering. Given the sharp decline in gene richness previously noted in this group, these results imply that the RL microbiota was in a state of ecological instability or collapse, failing to establish the coordinated and stable community structure observed in the ZL group (Figure 2A).

At the genus level, PCoA was employed to evaluate beta diversity, with PC1 (48.95%) and PC2 (17.95%) cumulatively explaining approximately 67% of the total taxonomic variance. During the early and middle developmental stages, assisted hatching did not induce significant structural shifts in the core microbiota. However, a pronounced divergence emerged in the late stage. The ZL samples exhibited high phylogenetic coherence, forming a tightly grouped cluster situated along the positive PC1 axis. Conversely, the RL group showed extreme spatial dispersion, with a confidence ellipse that encompassed a vast coordinate space, reflecting substantial inter-individual variability (Figure 2B). These patterns suggest that natural hatching promotes a highly convergent and stable community structure (ZL) in late-stage development, potentially representing an evolutionarily optimized gold standard for microbial assembly under natural selection. In contrast, assisted hatching appeared to introduce high levels of stochasticity into the successional trajectory. The marked heterogeneity within the RL group indicated that artificial intervention disrupted the canalized path of microbial succession, leading to an unstable and non-uniform ontogeny of the microbiota across individuals. These results implied that the natural hatching process—potentially involving exposure to eggshell fragments or parental oral secretions—provided a biological blueprint for coordinated microbial assembly. The tight clustering in the ZL group likely reflected the deterministic colonization of beneficial taxa, whereas the stochastic dispersion in the RL group suggested a shift toward neutral assembly or the sporadic colonization of opportunistic pathogens. Such microbial instability may ultimately under-lied the observed variance in health status and growth rates within the artificially hatched population.

**Figure 3:**
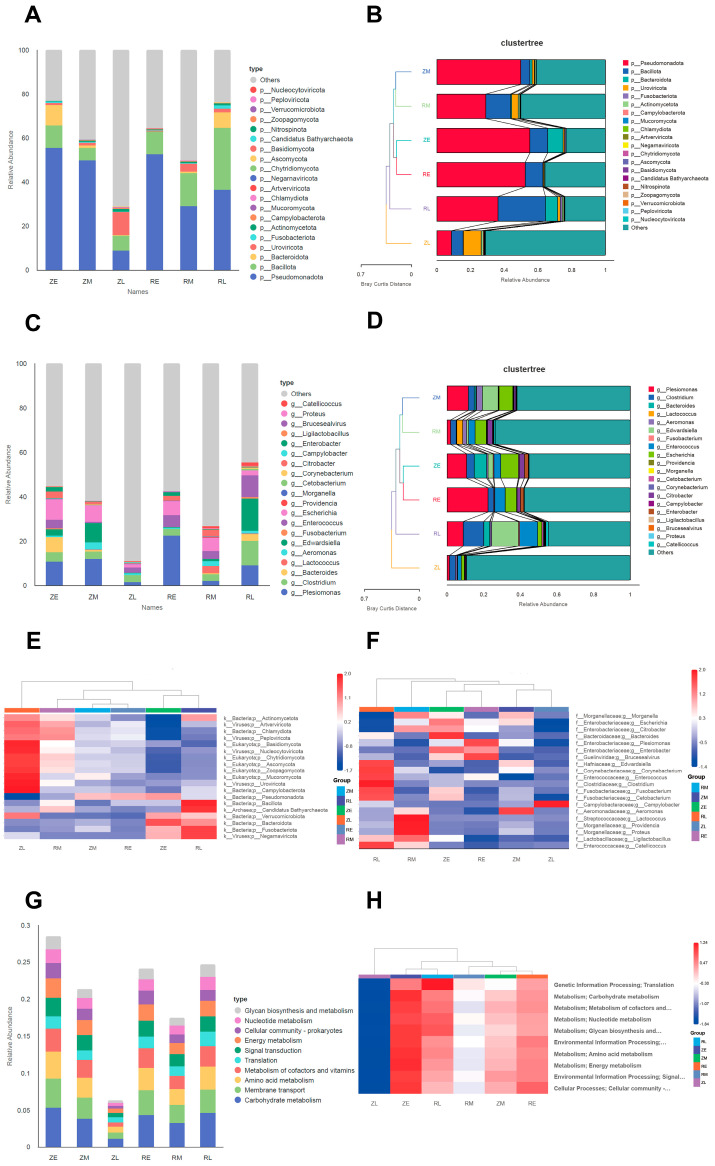
The proportions and differences in the bacterial communities in crested ibis: (**A**) Based on the species annotation results at the Phylum level, the top 20 species with the highest relative abundance for each group are selected, and the remaining species are set as “Others”. Bar charts of the relative abundance of species annotation results at different classification levels for each group are then plotted; (**B**) Clustertree of the top 20 species at the Phylum level; (**C**) Based on the species annotation results at the Genus level, the top 20 species with the highest relative abundance for each group are selected, and the remaining species are set as “Others”. Bar charts of the relative abundance of species annotation results at different classification levels for each group are then plotted; (**D**) Clustertree of the top 20 species at the Phylum level; (**E**) Cluster heatmap of the top 20 species at the Phylum level; (**F**) Cluster heatmap of the top 20 species at the Genus level; (**G**) The proportions of the top 10 KEGG functional categories; and (**H**) Clustertree of the top 10 KEGG functional categories.

At the phylum level, the ZM, RM, ZE, RE, and RL groups clustered together, indicating a high degree of similarity in microbial community composition among these cohorts. During the early and middle developmental stages, the natural (Z) and artificial (R) groups consistently exhibited close spatial proximity in the ordination; for instance, ZM and RM were positioned in extreme proximity, while ZE and RE also displayed high similarity. These observations align with the PCoA results, confirming that the hatching method did not significantly perturb the microbiota during the initial developmental phases. Across all groups except ZL, Pseudomonadota was the predominant phylum. However, its relative abundance declined sharply in the ZL group. Conversely, Bacillota exhibited a significant expansion in the RL group but remained at a comparatively low level in the ZL group. Notably, the proportion of taxa categorized as ‘Others’ in the ZL group was exceptionally high, exceeding 60%. This substantial contribution from minor taxa explains the elevated gene richness observed in the ZL group, which was characterized by a vast assemblage of low-abundance but highly diverse rare bacterial communities (Figure 3A,B, Table S1).

At the genus level, ZE and RE clustered together, indicating that at the time of development (the E stage), the taxonomic composition was highly similar regardless of the hatching method. Similarly, ZM and RM grouped together, suggesting that community succession patterns remained largely consistent through the middle stage of development. In contrast, the ZL group formed a distinct, isolated branch with a substantial branch length, indicating that the microbial structure of naturally hatched birds underwent a complete reconfiguration during the late stage. This mature state was significantly divergent from all other groups, including the late-stage artificial cohort (RL). Notably, the RL group failed to cluster with the ZL group and instead grouped with samples from the early and middle stages. This suggests that assisted hatching may cause microbial succession to stagnate at an intermediate state, preventing the community from evolving into the mature configuration observed in the natural group. *Plesiomonas*, an early dominant genus, declined rapidly over time in the Z cohort; however, this decline was attenuated in the R cohort, with the genus maintaining a significant presence in the RL group. Furthermore, *Clostridium* was significantly enriched in the RL group; members of this genus are frequently associated with intestinal environmental shifts or metabolic stress. *Edwardsiella* appeared to function as a transitional genus, showing higher proportions in both the ZM and RL stages. Finally, the top 20 genera accounted for less than 10% of the total composition in the ZL group, signifying an extremely diverse and evenly distributed community. This reinforced the findings from Figure 1A, confirming that the natural late-stage microbiota represented a highly diverse, stable system comprising numerous low-abundance rare taxa (Figure 3C,D, Table S2).

Clustering heatmaps at the phylum and genus levels were generated to evaluate community similarities. At the phylum level, the ZL group clustered independently on the far left, indicating that its taxonomic composition differed significantly from all other groups. Notably, the ZE group displayed high similarity to the RL group, with the two clustering together. This suggested that artificial shell removal led to a stagnation in microbiota development; even at the late stage (RL), the microbial structure remained characteristic of the early natural hatching stage (ZE) and failed to evolve into the mature ZL state. In the ZL group, taxa such as Actinomycetota, Uroviricota, and various eukaryotic phyla, including Ascomycota and Basidiomycota, exhibited significantly high abundances. As the organisms developed naturally toward the later stages, the community became increasingly complex, involving not only bacteria but also viruses and fungi in the formation of a stable micro-ecosystem (Figure 3E, Table S3). At the genus level, the RL group showed significantly high abundances of *Edwardsiella*, *Corynebacterium*, *Enterococcus*, *Clostridium*, *Fusobacterium*, and *Cetobacterium*. Several of these genera represented typical early intestinal colonizers or pioneer bacteria. For instance, *Enterococcus* is typically abundant during the initial days post-hatching. *Edwardsiella* was identified as a well-known opportunistic pathogen frequently associated with stress and intestinal inflammation. Regarding *Clostridium*, certain species (such as *Clostridium perfringens*) served as primary agents of necrotic enteritis in poultry. *Fusobacterium* functioned as a highly anaerobic pathogen often linked to mucosal damage, ulcers, and systemic inflammation. *Corynebacterium*, usually environment- or skin-derived, showed enrichment that indicated the physical barrier and competitive exclusion mechanisms of the intestine were not yet fully established. Furthermore, *Cetobacterium* was common in aquatic organisms but typically remained at low levels in the mature intestines of healthy terrestrial birds. The significant enrichment of these bacterial genera in the RL group, contrasted with the high-diversity stable state achieved by the ZL group, presented a clear negative biological signal. This indicated that the artificial shell removal intervention disrupted normal intestinal development, leading to altered environmental adaptation and metabolic plasticity in the young birds (Figure 3F, Table S4). Conversely, *Campylobacter* was the only genus among the top 20 that characterized the ZL group, where this curved bacillus functioned as a common symbiotic bacterium.

The top 10 functional categories predominantly involved essential survival and metabolic pathways, including carbohydrate, amino acid, and energy metabolism, alongside membrane transport and translation. Notably, the cumulative abundance of these housekeeping functions was significantly lower in the ZL group than in all other groups. This functional long-tail effect indicated that as chicks matured naturally, microbial roles shifted from general metabolism toward specialized secondary metabolism and niche-specific ecological regulation, signaling the establishment of a stable, mature climax community (Figure 3G, Table S5). In contrast, the RL group exhibited persistent developmental stasis, characterized by sustained high metabolic intensity. While functional abundance in the natural cohort followed a consistent downward trajectory (ZE > ZM > ZL), the artificial group displayed a rebounding U-shaped trend. Specifically, the RL group remained heavily engaged in basic nutrient metabolism—a hallmark of early-stage colonization—and failed to transition toward functional specialization. This resurgence in metabolic activity during the late stage (RL) was likely driven by the proliferation of opportunistic pathogens, which precluded the community from reaching the advanced successional equilibrium observed in natural hatchlings (Figure 3H, Table S6).

**Figure 4:**
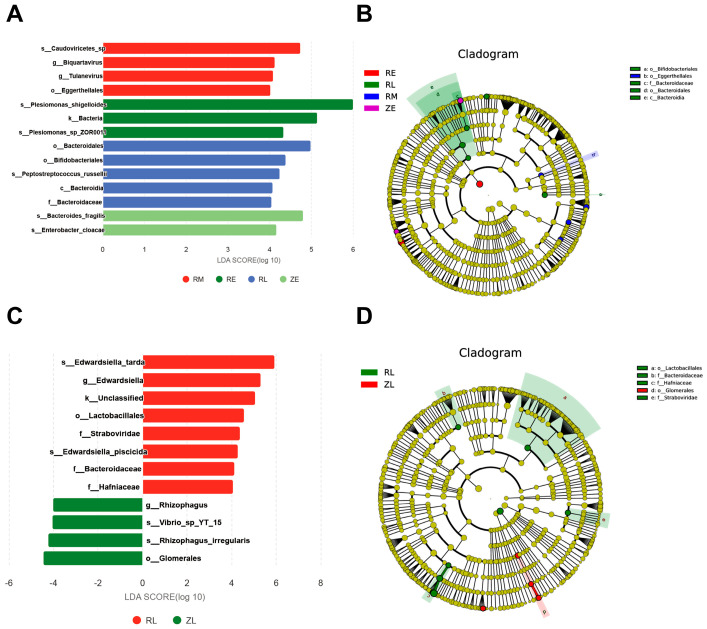
LEfSe analyses and evolutionary branching diagram of microorganisms: (**A**) LEfSe analyses of different groups. LDA (linear discriminant analysis) discriminant histogram. The LDA score obtained by LDA analysis indicates that the greater the LDA score, the greater the influence of species abundance on the difference effect; (**B**) Evolutionary branch diagram (unique phylogenetic distribution of species) and biomarkers with statistically significant differences between groups in the abundance comparison among different groups; (**C**) LEfSe analyses between the ZL and RL groups; and (**D**) Evolutionary branch diagram between the ZL and RL groups.

Significant biomarkers in the ZE group included *s_Bacteroides fragilis* and *s_Enterobacter_cloacae*, while the RE group was characterized by *s_Plesiomonas_shigelloides*. The latter yielded an LDA score of 6, indicating it functioned as the absolute core of the artificial cohort during the early stage. These results suggested that the hatching mode determined the initial dominant taxa; the natural group tended toward *Bacteroides* colonization, whereas the artificial group was occupied by *Plesiomonas*. Notably, the RM group exhibited a unique viral signature, with significant markers including *s_Caudoviricetes_sp*, *g_Biquartavirus*, and *g_Tulanevirus*. This enrichment of viruses and phages implied that artificial intervention triggered intense phage-bacteria confrontations, leading to substantial community instability during the middle stage. By the late stage, the RL group shifted toward a structure dominated by *o_Bacteroidales*, *o_Bifidobacteriales*, and *s_Peptostreptococcus_russellii*. In contrast, the ZL group displayed extremely high diversity and uniform species distribution (with ‘Others’ accounting for 90%). Because no single taxon exerted overwhelming dominance, the ZL group did not yield high-score biomarkers in the LEfSe analysis (Figure 4A). Cladogram analysis revealed that the evolutionary branches of the RL group were concentrated within the class Bacteroidia, order Bacteroidales, and family Bacteroidaceae (Figure 4B). This large-scale enrichment indicated that a functional system centered on anaerobic Bacteroidia was established in the RL group to degrade complex carbohydrates. Although Bifidobacteriales were also enriched in the RL group, their presence may reflect compensatory colonization under artificial intervention. Across all taxonomic levels, significant markers in the RL group included *s_Edwardsilella_tarda*, *g_Edwardsilella*, *k_Unclassfied*, *o_Lactobacillales*, *f_Straboviridae*, *s_Edwardsilella_piscicida*, *f_Bacteroidaceae*, *f_Hafniaceae* (LDA > 4). Conversely, the ZL group was significantly enriched with *g_Rhizophagus*, *s_Vibrio_sp_YT_15*, *s_Rhizophagus_irregularis*, *o_Glomerales* (Figure 4C). A comparative analysis of the evolutionary branches further confirmed these distinct phylogenetic distributions between the ZL and RL groups (Figure 4D).

### 3.2. Compositions and Differential Untargeted Metabolites in the Intestine of Crested Ibis

**Figure 5:**
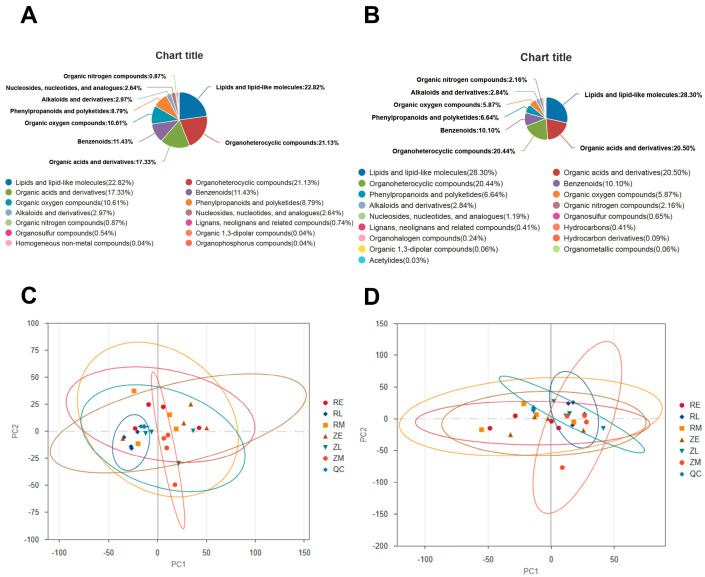
Metabolite classification of crested ibis fecal: (**A**) Based on the classify file database, all the metabolites identified in this project were annotated. The pie chart shows the anion mode classification and the proportion of metabolites contained in each classification (N = 4); (**B**) The pie chart shows the cationic mode classification and the proportion of metabolites contained in each classification; (**C**) PCA of the metabolites of cationic mode. ZE = early stage of natural hatching; ZM = middle stage of natural hatching; ZL = late stage of natural hatching; RE = early stage of artificial shell removal; RM = middle stage of artificial shell removal, RL = late stage of artificial shell removal); and (**D**) PCA analysis of the metabolites of cationic mode.

Untargeted LC-MS/MS analyses were conducted on fecal samples from the natural and artificial groups. In the anion mode, acidic metabolites produced by microbial fermentation or environmental sources were precisely captured. For instance, organic acids (such as lactic and succinic acid) containing carboxyl groups carried negative charges, while phospholipids, fatty acids, and nucleotides with phosphate groups exhibited high sensitivity. The top three metabolic categories were lipids and lipid-like molecules, organic heterocyclic compounds, and organic acids and derivatives, which collectively accounted for over 60% of the total. Lipids served as the primary substrates for yolk absorption, cell membrane synthesis, and energy storage; thus, lipid metabolism activity directly reflected the efficiency of nutrient utilization from the yolk. Organic heterocyclic compounds, including nucleotide bases, vitamins (such as biotin and riboflavin), and signaling molecules, indicated extensive genetic material processing and coenzyme synthesis. Organic acids and derivatives, including amino acids, tricarboxylic acid (TCA) cycle intermediates, and short-chain fatty acids (SCFAs), represented the key metabolic link between the microbiota and the host (Figure 5A). The cationic mode offered superior detection sensitivity for basic compounds, specific lipids (such as phosphatidylcholine), and certain amino acids. The top three metabolic categories were lipids and lipid-like molecules, organic acids and derivatives, and organic heterocyclic compounds. This mode detected a broader range of glycerophospholipids, sphingolipids, and free fatty acids, reflecting active yolk nutrient absorption and cell membrane assembly in the developing chicks (Figure 5B).

PCA was performed to assess the global metabolic profiles across all groups. In the anion mode, the PCA score plot revealed the distribution of the metabolomics data following dimensionality reduction. The confidence ellipses for the majority of the groups (RE, RM, ZE, ZM, and ZL) exhibited significant overlap, indicating that at the global acidic metabolic level, birds across different hatching methods and developmental stages shared similar basal metabolic substrates. The RL group showed a distinct leftward shift in sample distribution compared to the other cohorts and displayed tighter clustering. Notably, these metabolomic findings contrasted with the metagenomic PCoA results, where the ZL group was a prominent outlier due to its high diversity. In the metabolomic PCA, the divergence between the ZL group and other cohorts was less pronounced, reflecting functional redundancy between the microbiota and their metabolites. While the bacterial community structure in the late-stage natural group (ZL) underwent dramatic changes, these distinct assemblages likely performed similar metabolic functions, or the host maintained relatively stable metabolic homeostasis (Figure 5C). In the cationic mode, PCA presented the overall distribution of metabolites under positive ion monitoring. The quality control (QC) samples (light blue diamonds) were highly clustered in the center of the plot, confirming the stability and reproducibility of the analytical system. Similarly to the anion mode, the confidence ellipses of all experimental groups showed a significant range of overlap, suggesting consistent levels of metabolic homeostasis across the various developmental stages and hatching conditions (Figure 5D).

**Figure 6:**
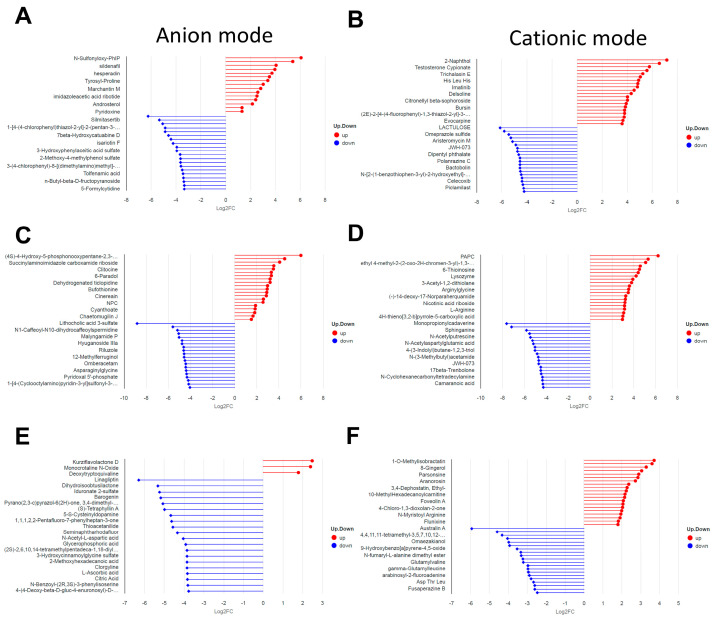
Stick diagram of differential metabolites between groups: (**A**) The differential metabolites between ZE vs. RE groups in the anion mode. The top 20 upregulated and downregulated metabolites, the color of the sticks is used to distinguish the upregulation (red) and downregulation (blue) of the metabolites, while the length of the sticks directly reflects the magnitude of the log2 (Fold Change) value, that is, the longer the stick, the greater the difference ratio, the size of the points represents the VIP value; (**B**) The differential metabolites between ZE vs. RE groups in the cationic mode; (**C**) The differential metabolites between ZE vs. RE groups in the anion mode; (**D**) The differential metabolites between ZM vs. RM groups in the cationic mode; (**E**) The differential metabolites between ZL vs. RL groups in the anion mode; and (**F**) The differential metabolites between ZE vs. RE groups in the cationic mode.

In NEG mode, down-regulated DMs predominated in the R groups compared to their Z counterparts; specifically, the number of down-regulated DMs increased from 62 in the early stage (ZE vs. RE) to 98 in the middle stage (ZM vs. RM), reaching a peak of 243 in the late stage (ZL vs. RL), while up-regulated DMs remained minimal (15, 22, and 3, respectively) (Figure 6A–C).

In POS mode, a similar trend of prevailing down-regulation was observed during the early and middle stages, with 136 and 159 down-regulated DMs recorded for the ZE vs. RE and ZM vs. RM comparisons, respectively. However, by the late stage (ZL vs. RL), the metabolic divergence became more balanced, with 52 up-regulated and 54 down-regulated DMs (Figure 6D–F). These results demonstrated that the metabolic disparity between hatching modes intensified as development progressed, particularly within the anionic metabolic pathways.

### 3.3. Metabolic Pathway Analysis

**Figure 7:**
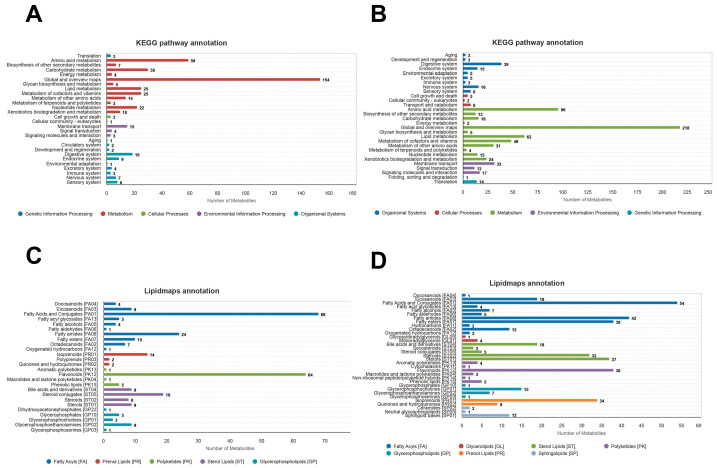
Top 20 KEGG pathway and lipid annotation analyses: (**A**) KEGG pathway annotation in the anion mode. The horizontal axis represents the number of metabolites, and the vertical axis represents the KEGG pathways annotated; this graph shows the number of metabolites annotated under each secondary classification within the Pathway primary classification; (**B**) KEGG pathway annotation in the cationic mode; (**C**) Lipid annotation in the anion mode; and (**D**) Lipid annotation in the cationic mode.

KEGG pathway annotation (anion mode) illustrated the functional distribution of compounds identified in the metabolomic analysis, providing a biological context for the physiological divergence between the natural (Z) and artificial (R) groups. The frequency and magnitude of the red bars—representing metabolic pathways—were significantly higher than those in other categories. This indicated that the core interaction between the microbiota and the host was centered on substance transformation and energy allocation. Within this category, the “Global and overview maps” (154 metabolites) covered central carbon metabolism, including glycolysis and the TCA cycle. The high abundance of these metabolites suggested that the chicks were in a phase of intense energy turnover, essential for the transition from yolk-derived nutrition to independent digestion. Amino acid metabolism (59 metabolites) ranked second, reflecting the robust protein synthesis and breakdown required for rapid growth, followed by carbohydrate metabolism (30 metabolites) involved in the degradation of complex polysaccharides (Figure 7A).

The cationic mode exhibited superior responses to basic compounds, amino acids, and specific phospholipids, identifying a greater total number of metabolites than the anionic mode. Consistent with the anionic results, the Metabolism category maintained absolute dominance. Specifically, Global and overview maps (218 metabolites), amino acid metabolism (96 metabolites), and lipid metabolism (62 metabolites) emerged as the most prominent functional sectors (Figure 7B).

Lipidmaps annotation (anion mode) provided a detailed secondary classification of lipid molecules, which served as the primary substrates for energy mobilization, membrane assembly, and signal transduction. Fatty acid derivatives predominated, particularly FA01 (Fatty Acids and Conjugates: 68 metabolites) and FA08 (Fatty Amides: 24 metabolites), with the latter often involved in neuroprotection and immune regulation. This high density of fatty acid molecules indicated that the analytical system successfully captured the intense lipid mobilization occurring in the developing chicks. Furthermore, the presence of PK12 (Flavonoids: 64 metabolites) suggested that the gut microbiota actively utilized plant-derived feed components to produce antioxidant or anti-inflammatory molecules. Variations in these metabolites between the RL and ZL groups potentially reflected differences in stress resistance. Additionally, ST05 (Steroid Conjugates: 19 metabolites) highlighted the complex endocrine regulation active during this stage (Figure 7C).

The cationic mode offered a more comprehensive characterization of lipids, particularly for nitrogen-containing bases and sterols. In this mode, Fatty Acid derivatives (FA01, FA02, and FA07) were identified as key players in lipid storage and turnover. Notably, the cationic mode captured a diverse array of Sterols (ST01: 37 metabolites) and Steroids (ST02: 32 metabolites); as precursors for developmental signals and cell membrane frameworks, these sterols reflected the endocrine and developmental status of the chicks. Moreover, the detection of Sphingolipids (SP01) and Glycerophosphocholines (GP01) underscored the critical roles of these lipids in maintaining intestinal barrier integrity and facilitating cell recognition (Figure 7D).

**Figure 8:**
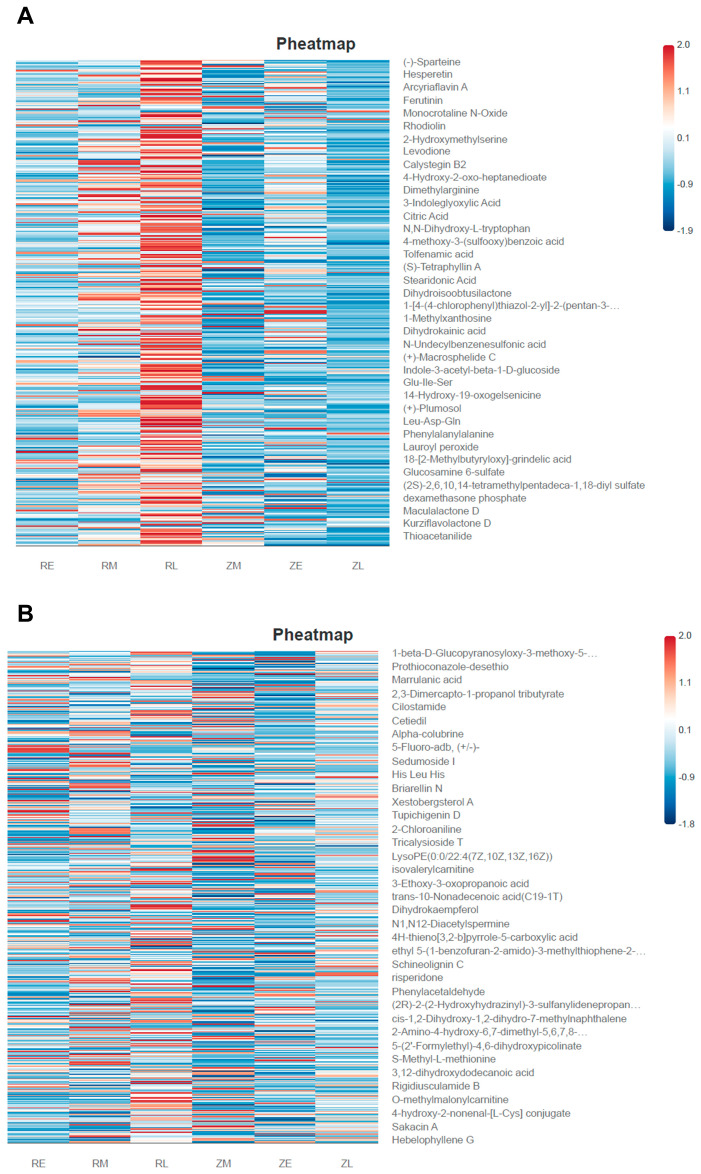
Heatmap of differential metabolites between groups: (**A**) Differential metabolites in the anion mode. After taking the union of the differential metabolites obtained from each comparison pair, hierarchical clustering analysis (HCA) was conducted. The relative quantitative values of all differential metabolites were subjected to scale transformation to eliminate the influence of different dimensions among variables. Based on these transformed data, hierarchical clustering analysis was performed to demonstrate the expression level differences in these differential metabolites among various groups; and (**B**) Differential metabolites in the cationic mode.

The metabolic heatmap revealed a distinct divergence in the late-stage development of the two groups. In the middle and lower regions of the plot, the RL group exhibited large, dense clusters of deep red, indicating a significant up-regulation of these metabolites. In sharp contrast, the ZL group displayed low abundance (dark blue) across the same regions. This divergence confirmed the “developmental arrest” or “metabolic deviation” hypothesis, suggesting that artificial intervention induced a chemical signaling profile entirely distinct from the natural state during late development. Among the key metabolites identified, organic acids, such as citric acid, were significantly increased in the RL group. As a core intermediate of TCA cycle, the abnormally high expression of citric acid in the RL cohort may indicate hypermetabolism or mitochondrial functional stress. Furthermore, lipids and unsaturated fatty acids, including stearidonic acid, were markedly elevated in the RL group. The accumulation of this Omega-3 unsaturated fatty acid likely corresponded to the intestinal inflammatory response triggered by the previously observed pathogenic conditions. Additionally, the abnormal increase in indole derivatives (e.g., indole-3-acetyl-beta-1-D-glucoside) in the RL group demonstrated that artificial shell removal altered the “microbiota-host” chemical dialog within the intestine. Peptides, such as Leu-Asp-Gln and phenylalanylalanine, also abounded in the RL group. The intestinal microbiota actively participated in the breakdown of dietary and endogenous proteins, utilizing bacterial proteases and peptidases to sequentially degrade large proteins into oligopeptides and free amino acids [8]. Commensal bacteria exhibited significant dipeptidyl peptidase-like activities, essential for cleaving bioactive peptides and regulating host metabolic pathways [9]. In this study, the altered structural assembly of the gut microbiome in RL chicks likely disrupted this proteolytic cascade. The accumulation of di- and tripeptides suggested that while initial protein cleavage occurred, subsequent microbial degradation into free amino acids was impaired or delayed. Rather than a definitive host absorption disorder, this metabolomic signature predominantly reflected a state of “microbial proteolytic dysregulation,” highlighting how hatching interventions indirectly altered nutrient bioavailability by shifting the metabolic function of the pioneer microbiome. While the natural group exhibited a stable and rhythmic successional migration, the artificial group underwent a metabolic “mutation.” The RE group remained metabolically similar to the ZE group (similar color distribution); however, by the RL stage, large clusters of unique metabolites emerged that did not overlap with the ZL profile. These findings indicated that while the influence of the hatching method remained latent during the early stages, it resulted in explosive biochemical consequences during late-stage development (Figure 8A).

Consistent with the findings in the anion mode, the cationic metabolite profile revealed the dynamic successional evolution across the developmental stages (E, M, and L). The cationic mode demonstrated superior sensitivity toward basic compounds, small peptides, and specific signaling molecules, thereby uncovering more granular biochemical divergence between the groups. Several key characteristic metabolites exhibited significant shifts. Specifically, the tripeptide His-Leu-His was identified in high abundance (deep red) within the RL group. As cationic detection is highly sensitive to peptide fragments, the extensive enrichment of such di- and tripeptides typically corresponds to excessive intestinal protein degradation or a host-mediated nutrient absorption disorder. Furthermore, Sakacin A was significantly increased in the RL group. As a bacteriocin produced by specific lactobacilli with known antimicrobial properties, its elevated expression suggested intense microbial competition within the RL group, potentially representing a physiological attempt to stabilize the dysbiotic community through the secretion of antibacterial substances. In the early and middle stages of the artificial group (RE and RM), alpha-colubrine (an α-Serpine-related compound) showed higher levels, which may have been linked to specific environmental exposures or stress responses unique to the artificial incubation setting. Finally, phenylacetaldehyde was enriched in the RL group. This compound is often a byproduct of phenylalanine metabolism, and its abnormal accumulation is frequently associated with the metabolic activities of intestinal pathogens, such as members of the *Enterobacteriaceae* family (Figure 8B).

**Figure 9:**
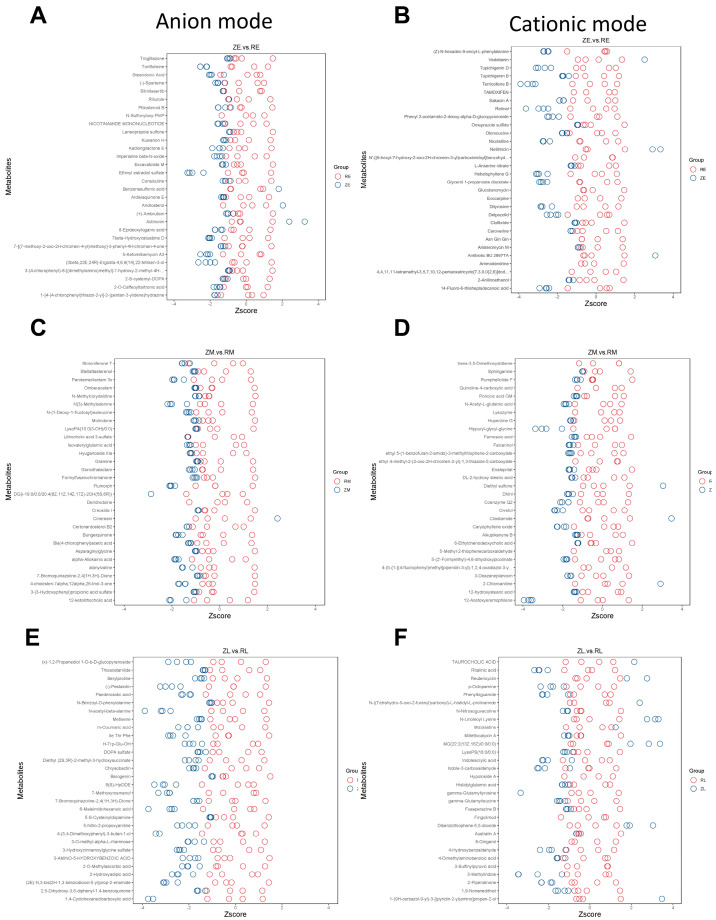
Z-score analysis of differential metabolites between groups: (**A**) Differential metabolites between the ZE and RE groups in the anion mode. The horizontal axis represents the transformed Z-values of the relative quantitative values of metabolites, and the vertical axis represents the names of the differential metabolites. Each circle represents a sample, where the red circles and blue circles, respectively, represent samples from two different groups. The top 30 most significantly different metabolites sorted by *p*-values from smallest to largest; (**B**) Differential metabolites between the ZE and RE groups in the cationic mode; (**C**) Differential metabolites between the ZM and RM groups in the anion mode; (**D**) Differential metabolites between the ZM and RM groups in the cationic mode; (**E**) Differential metabolites between the ZL and RL groups in the anion mode; and (**F**) Differential metabolites between the ZL and RL groups in the cationic mode.

Z-scores were calculated to normalize metabolite abundance across a common scale, facilitating the comparison of differential features between groups. Figure 9A,B illustrated the differential metabolites in both anion and cationic modes between the ZE and RE groups during the early stage. Similarly, Figure 9C,D presented the metabolic divergence between the ZM and RM groups in the middle stage. Finally, the late-stage shifts between the ZL and RL groups were summarized in Figure 9E,F (Figure 9A–F).

**Figure 10:**
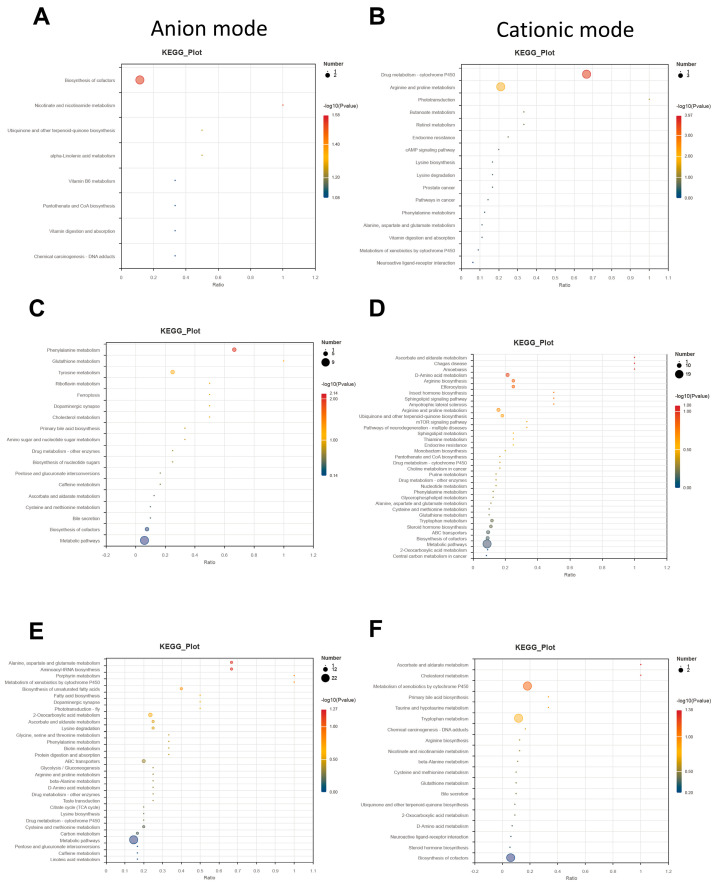
Top 20 KEGG enrichment analyses: (**A**) Top 20 KEGG enrichment analysis between the ZE and RE groups in the anion mode (*p*-value method). The horizontal coordinate is the enrichment significance *p*-value; generally, the size of the bubbles in the figure represents the amount of concentration of metabolic compound in this pathway; (**B**) Top 20 KEGG enrichment analysis between the ZE and RE groups in the cationic mode; (**C**) Top 20 KEGG enrichment analysis between the ZM and RM in the anion mode (*p*-value method); (**D**) Top 20 KEGG enrichment analysis between the ZM and RM in the cationic mode; (**E**) Top 20 KEGG enrichment analysis between the ZL and RL groups in the anion mode (*p*-value method); and (**F**) Top 20 KEGG enrichment analysis between the ZL and RL groups in the cationic mode.

Pathway enrichment analysis was conducted to identify the primary metabolic shifts between the artificial and natural hatching groups. In the early stage (ZE vs. RE), the most significant pathways included the biosynthesis of cofactors, vitamin digestion and absorption, neuroactive ligand-receptor interactions, and the metabolism of xenobiotics by cytochrome P450 (Figure 10A,B). During the middle stage (ZM vs. RM), metabolic pathways and the biosynthesis of cofactors emerged as the dominant enriched categories in both anion and cationic modes (Figure 10C,D). Finally, the late-stage comparison (ZL vs. RL) revealed significant enrichment in metabolic pathways, carbon metabolism, biosynthesis of cofactors, and steroid hormone biosynthesis (Figure 10E,F).

### 3.4. Correlations Between Intestinal Microbes and Metabolites

**Figure 11:**
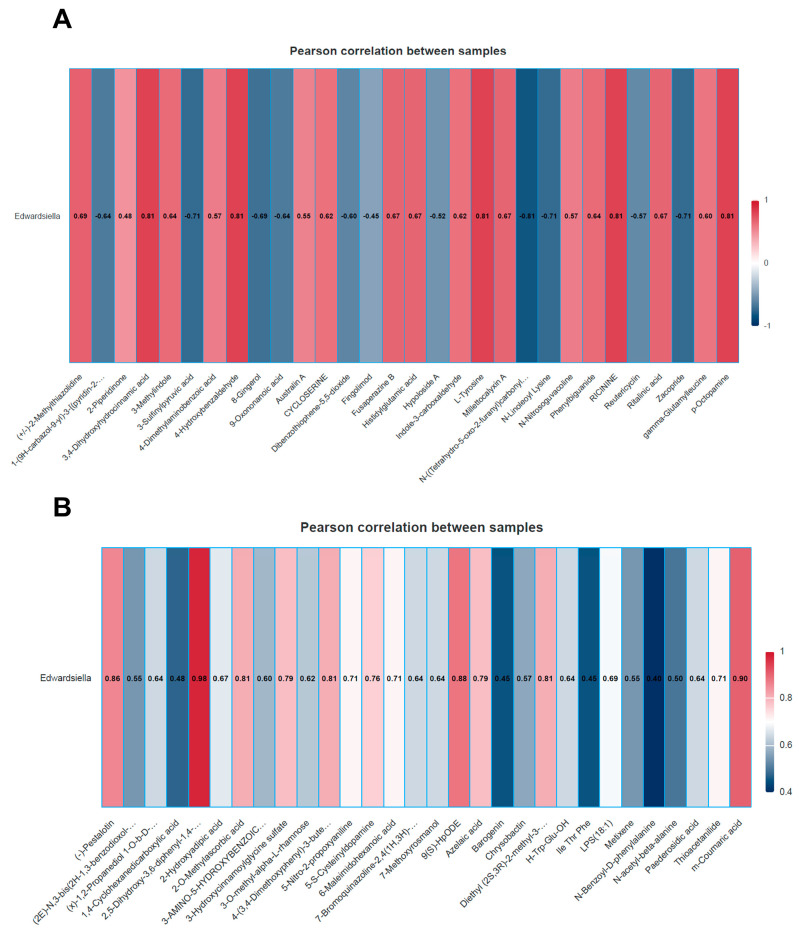
Differential microbial and differential metabolite association analysis: (**A**) The microorganism and metabolite association analysis in the anion mode. Based on the Spearman correlation coefficient, a correlation analysis was conducted between the significantly different bacterial communities identified by microbial analysis and the significantly different metabolites obtained from metabolomics analysis. When the changes in metabolites are in line with those of a certain bacterial community, they show a positive correlation; conversely, if the trends are opposite, they exhibit a negative correlation; and (**B**) The microorganism and metabolite association analysis in the cationic mode.

Pearson correlation analyses were performed at the genus level to evaluate the relationships between the microbiota and the metabolome. In the comparison between the ZL and RL groups, *g_Edwardsilella* was identified as the sole significant microbial marker characterizing the RL group. In the cationic mode, *g_Edwardsilella* showed significant correlations with several metabolites, including L-Tyrosine, 3,4-Dihydroxyhydrocinnamic acid, 4-Hydroxybenzaldehyde, RICININE, p-Octopamine, N-Linoleoyl Lysine, Zacopride, 3-Sulfinylpyruvic acid, and N-((Tetrahydro-5-oxo-2-furanyl)carbonyl) -L-histidyl-L-prolinamide (Figure 11A, Table S7). Similarly, in the anion mode, this genus was significantly correlated with 5-Nitro-2-propoxyaniline, Thioacetanilide, 9(S)-HpODE, 6-Maleimidohexanoic acid, (-)-Pestalotin, 2,5-Dihydroxy-3,6-diphenyl-1,4-benzoquinone, m-Coumaric acid, Azelaic acid, 4-(3,4-Dimethoxyphenyl)-3-buten-1-ol, 2-O-Methylascorbic acid, 3-Hydroxycinnamoylglycine sulfate, 5-S-Cysteinyldopamine, and Diethyl (2S,3R)-2-methyl-3-hydroxysuccinate (Figure 11B, Table S8).

## 4. Discussion

As crested ibis conservation transitions from “preserving quantity” to “improving quality,” evaluating the long-term biological costs (trade-offs) of non-natural interventions is essential. While assisted hatching can increase hatching rates, its impact on the intestinal health and metabolic profiles of rare birds remains poorly understood. This study compared the gut microbiota and metabolic signatures of naturally hatched individuals against those subjected to assisted hatching. These findings facilitate a scientific evaluation of current hatching techniques and provide quantitative molecular indicators for optimizing artificial rearing—such as the development of probiotic-enriched formulas—and improving the selection of individuals for wild release.

Artificial shell removal—which produces shell-less or semi-shell-less embryos—indirectly influences the gut microbiota by altering embryonic colonization and host physiology, particularly calcium metabolism, rather than through a direct antimicrobial effect [10]. Given that newly laid eggs are nearly sterile, the intestinal microbiota is primarily established post-oviposition via the eggshell, nest environment, parents, and feed. The eggshell surface, harboring maternal and environmental bacteria, serves as a critical vector for “founder” species that inoculate the chick during hatching [11].

Metagenomic diversity within the gut dictates the functional repertoire and resilience of the microbial ecosystem, ultimately influencing host nutrition, immunity, and disease susceptibility [12]. A diverse microbial gene pool supports essential processes, including carbohydrate fermentation, energy production, and the synthesis of cellular components, thereby optimizing energy extraction from the diet. Furthermore, the synthesis of critical metabolites, such as short-chain fatty acids (SCFAs) and vitamins, relies on a broad array of metabolic genes distributed across diverse taxa [13]. This extensive genetic reservoir also facilitates the modification of bile salts and the degradation of xenobiotics, which collectively enhance the survival rates of juvenile birds.

Gut microbiota plays a pivotal role in animal health and production performance [14,15]. In this study, taxonomic analysis of crested ibis feces revealed that the abundances of Pseudomonadota and Bacillota were significantly elevated in the RL group compared to the ZL group. Members of the diverse Pseudomonadota phylum contribute to global carbon, nitrogen, and sulfur cycles through processes such as ammonia oxidation and organic compound degradation [16]. Similarly, the Bacillota phylum encompasses a wide range of ecological roles; many of its members, including *Bacillus*, *Clostridium*, and *Paenibacillus*, are instrumental in decomposing complex plant polymers like cellulose and pectin [17]. The metabolic flexibility of Bacillota—ranging from aerobic to strict anaerobic lifestyles—allows these bacteria to occupy diverse niches within the avian gut [18]. Notably, the proportion of “Other” taxa in the ZL group exceeded 60%, which likely accounted for the superior gene counts observed in the natural cohort. This high prevalence of unclassified or low-abundance taxa indicated that the ZL group harbored a highly diverse rare bacterial communities.

At the genus level, the RL group failed to cluster with the ZL group, instead aligning with samples from the early and middle stages. This suggested that artificial shell removal induced a stagnation in bacterial succession, preventing the community from maturing into the state observed in the natural group. This developmental arrest was characterized by a significant enrichment of *Plesiomonas*, *Clostridium*, and *Edwardsiella* in the RL group.

In avian species, *Plesiomonas* is primarily regarded as an opportunistic pathogen rather than a beneficial commensal. *Plesiomonas shigelloides* has been identified as a causative agent of enteric disease and sepsis in waterbirds [19]. Surveys indicated that birds act as major intestinal reservoirs for this bacterium, with high fecal prevalence reported in several species [20,21]. Furthermore, *Plesiomonas* isolates from crested ibises frequently exhibit multi-drug resistance, raising concerns regarding the dissemination of resistant strains within protected populations.

The genus *Clostridium* in the avian gut encompasses both beneficial commensals (*Clostridium butyricum*) and important pathogens (*Clostridium perfringens*) [22]. While butyrate-producing species like *Clostridium butyricum* enhance the intestinal barrier and nutrient utilization [23,24], *Clostridium perfringens* is a primary driver of necrotic enteritis, leading to mucosal lesions and increased mortality [25,26]. Similarly, *Edwardsiella* is considered a transient microbe or opportunistic pathogen carrier in birds, often acquired through contaminated water or prey [27]. There is no evidence suggesting *Edwardsiella* contributes to fermentation or immune modulation; instead, it poses a risk for systemic edwardsiellosis during periods of intestinal inflammation or immune compromise [28,29]. This shift toward a more pathogenic profile in the RL group, marked by the concurrent enrichment of *Clostridium perfringens* and *Edwardsiella*, further highlighted the metabolic and immunological instability triggered by artificial hatching.

In the ZL group, the top 20 genera accounted for less than 10% of the total relative abundance, indicating an exceptionally diverse and evenly distributed microbial community. This affirmed the findings from Figure 1A, where the natural late-stage cohort exhibited the highest gene counts, supported by a mature and stable ecosystem comprising numerous low-abundance, rare microbial taxa. Although these shifts in the RL group were not accompanied by acute clinical symptoms during the 33-day monitoring period, this physiological tolerance likely stemmed from the highly controlled and sanitized conditions of artificial rearing. However, we interpreted this structural shift not as a benign adaptation, but as a state of subclinical vulnerability. For instance, Edwardsiella tarda is a documented opportunistic pathogen that often remains dormant until triggered by environmental stress. This pathogen-permissive microbial baseline may lower the immunological threshold of RL individuals, potentially increasing susceptibility to systemic infections during subsequent growth phases or upon reintroduction into the wild.

A critical question remains whether the microbial stagnation observed in the RL group possesses the resilience to recover as the birds mature. While ontogenetic dietary shifts—such as the transition to a wild-type diet—can dramatically reshape the microbiome, the ecological principle of “priority effects” suggests that the timing of early colonization profoundly dictates the trajectory of host immune education. The initial 33 days post-hatching represent a critical window for the maturation of gut-associated lymphoid tissue. Therefore, even if the RL microbiome structurally recovers later in life, the functional legacy of early-stage stagnation may persist. This developmental deficit could manifest as compromised gut barrier integrity or altered immune responses, rendering individuals more susceptible to environmental pathogens. Consequently, while assisted hatching ensures immediate survival, longitudinal studies extending into sub-adult phases are imperative to determine if early microbial stagnation translates into long-term fitness costs—a vital consideration for optimizing pre-release evaluations in crested ibis conservation programs.

LEfSe analysis was employed to identify high-dimensional biomarkers characterizing the divergence between hatching modes. At the late stage, *Edwardsiella* emerged as the sole significantly enriched genus in the RL group. Integrated correlation analysis between metagenomic and metabolomic data further revealed that this enrichment was closely linked to perturbations in neuroactive signaling, sulfur amino acid catabolism, and lipid peroxidation.

In the cationic mode, p-octopamine showed a significant positive correlation with *Edwardsiella* and was involved in the neuroactive ligand-receptor interaction pathway. As a biogenic amine neurotransmitter, octopamine modulates neural excitability through specific G protein-coupled receptors [30,31]. While its enteric role in avian species remains to be fully defined, its enrichment suggests a potential modulation of gut neural circuits or smooth muscle tone in RL individuals [32,33]. Conversely, 3-sulfinylpyruvic acid displayed a negative correlation with *Edwardsiella*, involving cysteine and methionine metabolism. This metabolite represents a critical link between sulfur amino acid catabolism and energy metabolism (via pyruvate) [34,35]. The reduced levels of 3-sulfinylpyruvic acid in the RL group may indicate a diminished flux through the transsulfuration pathway, potentially impacting redox balance and epithelial turnover, which are essential for maintaining intestinal homeostasis [34,36].

In the anion mode, 9(S)-HpODE—an oxidized derivative of linoleic acid—exhibited a positive correlation with *Edwardsiella*. The enrichment of 9(S)-HpODE, alongside active linoleic acid metabolism, points to heightened oxidative stress and inflammatory signaling [37,38,39]. In the avian gut, peroxidized lipids like 9(S)-HpODE can trigger pro-inflammatory cascades and impair barrier integrity, reflecting a compromised intestinal environment in RL chicks [37,40]. Furthermore, the positive correlation with m-coumaric acid (a microbial phenolic metabolite) underscores alterations in phenylalanine metabolism. In birds, phenylalanine processing involves complex interactions between host absorption and microbial transformation [41,42]. The accumulation of m-coumaric acid in the RL group serves as a metabolic signature of how the stagnant microbiota processes aromatic substrates, further reflecting the altered redox and inflammatory state induced by artificial shell removal [41].

The significant gut microbiota variations observed between hatching methods align with studies in domestic poultry (*Gallus gallus*), where artificial incubation often leads to delayed maturation and Proteobacteria dominance due to the lack of maternal contact [43]. However, the artificial eggshell peeling performed in endangered species conservation represents a more extreme mechanical intervention than standard artificial incubation. During a natural avian hatching process, an embryo spends several hours to days ‘pipping’, a process that involves the direct oral ingestion of amniotic fluid, eggshell membranes, and shell fragments. Recent studies have demonstrated that the avian eggshell acts as a critical transgenerational carrier of maternal skin, feather, and nest-soil microbiomes [44]. By manually extracting the crested ibis chick to prevent exhaustion, conservationists inadvertently bypass this crucial oral-eggshell microbial exposure window. While previous research on the crested ibis has established that the first month of life is a highly dynamic period for normal microbial succession and host growth [45], our findings provide novel evidence that the physical method of hatching fundamentally alters the starting point of this succession. Unlike domestic poultry studies which primarily attribute microbial shifts to the sterile incubator environment, our comparative data suggests that the mechanical absence of the pipping and shell-breaking process itself deprives the artificial-peeling group of foundational pioneer taxa, thus altering the evolutionary trajectory of the gut flora from days 3 to 33 post-hatching.

For a species recovering from an extreme genetic bottleneck like the crested ibis, maximizing the sheer number of surviving offspring is the immediate priority. Artificial eggshell peeling is an emergency intervention that saves weak embryos that would otherwise perish during the exhausting hatching process. Abruptly halting this practice would cause an immediate and sharp increase in embryonic mortality. As the crested ibis population fully stabilizes and expands in the future, conservation programs could gradually phase out artificial interventions. We also could swab the eggshells of naturally incubating birds, or take cloacal swabs from healthy adults, and gently apply this to their initial food sources. This mimics the vertical transmission they miss out on when stripped from the shell. During the critical window of gut microbiota evolution (days 3 to 33), the artificial diet should be supplemented with species-specific beneficial probiotics. Supporting the early proliferation of foundational bacteria helps stabilize the gut flora, accelerates the maturation of the mucosal immune system, and outcompetes opportunistic environmental pathogens.

Limitations of the Study. Our functional interpretations are based on DNA-level metagenomic predictions and metabolomic profiling, lacking direct in vivo validation. Due to the critically endangered status of the crested ibis, invasive physiological sampling and manipulative experiments are ethically and legally prohibited. Furthermore, to minimize handling stress, longitudinal phenotypic tracking was not conducted. Consequently, the identified metabolic shifts and metabolite accumulations should be interpreted as evidence of metabolic plasticity or developmental divergence rather than confirmed pathological disorders. Future research should integrate these molecular baselines with non-invasive physiological monitoring and metatranscriptomics to better understand the long-term fitness costs of early microbial stagnation.

## 5. Conclusions

This study demonstrates that artificial eggshell peeling imposes significant negative impacts on the successional trajectory and metabolic maturation of the gut microbiota in crested ibis chicks. While natural hatching facilitates the assembly of a highly diverse, functionally specialized, and stable mature microecosystem (ZL group), artificial intervention induces developmental stasis and community instability. Specifically, the RL group exhibited a marked reduction in genetic diversity and a significant enrichment of opportunistic pathogens, including *Edwardsiella*, *Clostridium*, and *Fusobacterium*. Metagenomic and metabolomic analyses further confirmed a pronounced metabolic deviation in the artificial intervention group. These individuals maintained high-intensity basal metabolism characteristic of early colonization into their later developmental stages, leading to the accumulation of metabolites associated with mitochondrial dysfunction (e.g., abnormal citric acid elevation), intestinal inflammation, and excessive protein degradation. The strong correlation between the core pathogen *Edwardsiella* and multiple aberrant metabolites underscores the systemic risks of this hatching method. Ultimately, while ensuring immediate survival, artificial shell peeling triggers pathological developmental retardation and immune–metabolic distress, highlighting the urgent need for optimized microbial restoration strategies in the conservation of this endangered species.

## Data Availability

Data are available from the corresponding author upon reasonable request.

## Supplementary Materials

The following supporting information can be downloaded at: https://www.mdpi.com/article/10.3390/ani16101472/s1, Table S1. The proportions of the bacterial communities at the Phylum level. Table S2. The proportions of the bacterial communities at the Genus level. Table S3. The top 20 species at the Phylum level. Table S4. The top 20 species at the Genus level. Table S5. The proportions of the top 10 KEGG functional categories. Table S6. The top 10 KEGG functional categories. Table S7. The microorganism and metabolite association analysis in the anion mode. Table S8. The microorganism and metabolite association analysis in the cationic mode.
